# American Indian Motherhood and Historical Trauma: Keetoowah Experiences of Becoming Mothers

**DOI:** 10.3390/ijerph19127088

**Published:** 2022-06-09

**Authors:** December Maxwell, Rebecca Mauldin, Johanna Thomas, Victoria Holland

**Affiliations:** 1 Thompson School of Social Work & Public Health, University of Hawaii at Manoa, Honolulu, HI 96822, USA; 2 School of Social Work, The University of Texas at Arlington, Arlington, TX 76019, USA; rebecca.mauldin@uta.edu; 3 School of Social Work, University of Arkansas at Fayetteville, Fayetteville, AR 72701, USA; johannat@uark.edu; 4 United Keetoowah Band, Tahlequahd, OK 74465, USA; proctorvs@gmail.com

**Keywords:** perinatal mental health, cultural, social and structural determinants of health and equity, historical trauma, story inquiry, maternal child health, culturally safe and responsive health services

## Abstract

Background: American Indian/Alaskan Native (AI/AN) women disproportionally experience postpartum depression in the United States as compared to the rest of the population. Despite being disproportionately represented, the current body of knowledge lacks research on depression in this particular population. Specifically, the current literature lacks research pertaining to the experiences of postpartum AI/AN women, their culture, birthing and mothering expectations, and trauma. This qualitative study used the theories of becoming a mother, historical-trauma framework, and reproductive justice as they relate to Indigenous women’s personal and historical trauma to assess their lived experiences of becoming a mother. Methods: Keetoowah mothers (*N* = 8) were interviewed by using a story inquiry method to understand the perinatal experiences of members of one Indigenous tribe in the US. Findings: The story inquiry coding resulted in two main themes, namely maternal mental health challenges and inadequacies of perinatal care. Conclusion: The subthemes illuminate the intersection of historical trauma and the perinatal experience, continued colonization of mothering, and the resilience of tribal culture during the postpartum period. Implications include advocacy for increasing culturally derived perinatal interventions, increased healthcare coverage of culturally appropriate birthing practices, and future research evaluating the correlation between historical trauma and maternal mental health challenges.

## 1. Introduction

In the United States, 3.8 million births occur annually, 30,000 of which are American Indian/Alaskan Native (AI/AN) mothers [1]. Approximately one in eight US mothers will experience postpartum depression (PPD) [2]. Globally, the prevalence of PPD is drastically different based on population size. For example, 12% of Indigenous women in Canada will experience PPD [3], 17% among AI/AN women [4], and more than one-third (38%) among low-income Hispanic mothers [5]. Among Pakistani mothers, nearly two-thirds (63%) experience PPD [6]. These rates indicate a broad range of PPD rates and also highlight the racial and ethnic disparities in PPD prevalence.

Postpartum depression (PPD) is a major depressive disorder. The Diagnostic and Statistical Manual of Mental Disorders defines PPD as depressive symptoms that follow a child’s birth. Symptoms often include apathy; a lack of interest in the baby; feeling anxious about the baby or a lack of bonding; feelings of being a bad mother; feeling hopeless, worthless, and sad; and even having suicidal thoughts and ideations [7]. PPD may also include anxiety around thoughts of harming the baby either intentionally or unintentionally (e.g., shaking the baby, falling with the baby, and car accidents) [8]. PPD often affects new mothers’ ability to socialize and find necessary supports, as the depressive symptomology often comes with a reduction in interest in these beneficial activities [9]. Healthcare expenditures for women with PPD are approximately 90% more than for non-depressed women [10]. Moreover, the leading cause of maternal death one year post-childbirth is suicide [11], and suicide, in general, among AI/AN women is 3.5 times higher than for any other racial and ethnic group of women in the US [12].

One contributing factor to developing PPD is the experiences a mother has as she becomes a mother. The becoming a mother (BAM) theory explains the many concepts and dimensions of the transition into motherhood. This transition can be shaped by a multitude of personal and societal factors; cultural context also contributes to how women become mothers, ultimately affecting maternal mental health. 

Even though PPD affects mothers of all ethnicities and socioeconomic backgrounds, there are populations that are disproportionately affected. American Indian/Native American (AI/AN) mothers have much higher rates of PPD, and research investigating unique epidemiological reasons for this exists but is not exhaustive [13]. The way that AI/AN mothers experience the role transition to motherhood, or BAM, is one possible experience related to the development of PPD for this population. As such, this qualitative study centered on the BAM experience for one tribe, the Keetoowah, through a lens of reproductive justice and historical trauma.

### 1.1. Theoretical Approach

Becoming a mother (BAM) is a construct that encompasses life-changing transitions into the maternal role, such as expectations of motherhood and any conflicts in the maternal role [14]. BAM theory [15] has identified several relevant concepts and dimensions of the transitional process from non-motherhood into motherhood, which is shaped by many personal, cultural, and societal factors affecting maternal mental health [9]. BAM theory suggests that BAM beliefs influence many aspects of maternal life, including (1) the birthing process, (2) concerns about pregnancy, (3) attachment to the fetus, (4) maternal-role attainment, (5) beliefs about maternal capability, (6) devotion to motherhood, (7) self-confidence as a mother, (8) maternal role flexibility, (9) security in maternal identity, (10) attitudes toward childrearing, (11) maternal role strain, and (12) experiences within the postpartum period [15]. 

A mother’s orientation toward BAM can rely on cultural mothering expectations, and incongruence with cultural expectations can strongly influence maternal mental health [16,17,18]. Furthermore, marginalized populations have experienced BAM differently due to social and political inequalities relating to reproductive justice [19]. Experiences with historical–cultural erasure; forced relocation; racist policies that control mothering, such as forced sterilization and limited birth control access; and racial profiling among child welfare policies have altered the BAM experience for AI/AN women [20,21]. 

Historical trauma is a potential unique risk factor for PPD among AI/AN women, influencing the BAM experience. Historical trauma is defined as a historically traumatic event experienced by a specific body of people. The effects of historical trauma can be traced intergenerationally and are often widespread in the community [22,23]. It encompasses emotions and actions resulting from the historically traumatic event, including grief resolution, lower self-esteem, depression, anxiety, and anger [23]. Current research indicates that historical trauma is linked to a plethora of mental and social health concerns such as substance abuse [24], intimate partner violence (IPV) [25], and somatic expressions of PPD among AI/AN women [26]. 

The historical context of reproductive justice specifically influences BAM for AI/AN mothers and may contribute to the uncommonly high rates of PPD pervasive among AI/AN mothers. History shows that several reproductive injustices perpetuated against AI/AN women connect to historical trauma. The reproductive justice framework incorporates the intersectional impacts of economic injustice and social and political inequalities (e.g., historical trauma) that contribute to inequitable reproductive rights of marginalized groups of people [27]. Notably, reproductive justice includes the right to have children and the “conditions under which one gives birth, and the ability to parent children with support, safety, and dignity” (p. 693, Reference [28]). Historically, motherhood for AI/AN women in the US was controlled by colonizers through forced abortions, forced or coerced sterilization, and by limiting or restricting abortion and birth control access [20,21]. Although cesarean deliveries constitute approximately one-third of all births in the US, AI/AN are much more likely to deliver via cesarean than other minority groups. This is especially prevalent among AI/AN women with lower educational status even when controlling for other health risks and potential prenatal complications. Together, this indicates a higher rate of non-medically necessary cesarean delivery [29]. Lastly, the ability to parent children with support, dignity, and safety has been repeatedly jeopardized for AI/AN mothers because AI/AN mothering has been repeatedly criminalized via racist child welfare policies and tactics, including forced removals or adoptions of children [30]. 

Due to the relevance of culture to BAM and the distinct and complex history of AI/AN people, this paper looks beyond the documented biological and mental health risk factors for PPD [31,32] to focus on BAM experiences and maternal mental health through the theoretical lens of becoming a mother (BAM). We acknowledge that reproductive injustices and other historical contexts, such as forced removal, cultural erasure, and disenfranchisement, are likely interwoven into the unique challenges of BAM for AI/AN women, and by extension, PPD [19,33,34]. These intersections lend to the chosen theoretical approach to AI/AN experiences of BAM used in this study. 

### 1.2. Empirical Evidence Linking Becoming a Mother (BAM) and Postpartum Depression

Attitudes and experiences of BAM are related to PPD development in a multitude of ways, psychologically, socially, and spiritually. Psychologically, having positive regard to maternal identity and maternal roles, feeling attached to their infant, and satisfaction with life changes related to motherhood are related to enhanced mental health and lower PPD risk among mothers [35,36,37]. Similarly, maternal confidence during the transition to motherhood is positively associated with maternal mental health outcomes [38]. In contrast, dysfunctional beliefs toward mothering, such as feeling inept, having disproportionately negative views about maternal competence, or negatively perceiving the postpartum period, are negatively related to PPD [18,36]. Socially, aspects of BAM, including support during labor by other women, can reduce negative feelings about childbirth [39] that are associated with elevated PPD risk. Conversely, perinatal care providers’ lack of support is linked to an increased risk of postpartum mental health challenges [40]. Religious or philosophical orientation can connect maternal identity to spirituality, and a lack of such an orientation is tied to lower maternal confidence and increased risk of PPD [41]. As it applies to the maternal transition, spirituality has acted as a buffer to postpartum mental health challenges and acted as a coping mechanism when a mother is diagnosed with PPD [42]. Even more logistical aspects of BAM can be related to PPD. For example, birth-plan disruption is also associated with an increased risk of PPD [40].

### 1.3. Cultural Differences in Becoming a Mother

There are some shared or universal aspects of BAM, such as embracing a new maternal identity, physical changes of a woman’s body, and the adjustment that comes with adding a baby to the family dynamics (Evans et al. 2012). However, culturally imposed roles and expectations may create vastly different BAM experiences. The literature on various cultural differences regarding BAM is rich, and we highly recommend such works for the interested reader [43,44,45,46,47,48,49,50,51,52,53,54,55]. Specifically, the literature centers on cultural differences in maternal self-identity and confidence [43,45,49,55], meaning of childbirth [44,47,51,52,54], maternal mental health [48,50,53], and motherhood practices, including spirituality [46,53]. The literature demonstrates that beliefs such as whether motherhood is a woman’s destiny, that birthing ability is tied to maternal strength, or that enduring labor is a rite of passage vary by culture. So too does the association of mother’s self-identity and cultural belonging with giving birth, or giving birth in culturally ideal ways (e.g., vaginally, alone, or with others for support). As with all aspects of fertility, pregnancy, and birthing, there are vast cultural variations in the meaning ascribed to difficulty with birthing or depression and other mental health concerns that follow a child’s birth. Often, a mother’s sense of maternal competence depends on cultural values and expectations. All aspects of BAM are dependent upon a mother’s cultural background, from preparation during pregnancy to birthing rituals to postpartum practices. Understanding the cultural expectations of the maternal role for women when addressing PPD can deepen the understanding of PPD etiologies since incongruence with cultural expectations of BAM can lead to anxiety and diminished maternal competence and self-esteem [34].

### 1.4. Becoming a Mother (BAM) among Keetoowah and Cherokee People

The current study focuses on BAM experiences and the maternal mental health of the AI/AN Keetoowah tribe (The United Keetoowah Band Tribe was consulted on the findings of this paper and have approved all stories, findings, quotes, and other information pertaining to the tribe. In addition, the tribal council assisted in the findings related to policy and research implications for their tribe). The Keetoowah, traditionally known as Giduwah, and contemporarily known as the United Keetoowah Band (UKB), has approximately 14,000 members and is the eighth largest tribe in Oklahoma. Although the UKB, the Cherokee Nation of Oklahoma, and the Eastern Band of Cherokee Indians are all made up of Cherokee people, they are recognized by the US as being separate sovereigns. Each of the aforementioned tribes shares a common history, as well as overlapping cultural values and traditions as Cherokee people. The UKB relocated from the southern region of the US, near present-day Georgia, after the vast majority of their land was taken in treaty agreements. These treaties forcibly moved the UKB during the Trail of Tears [56,57]. As an involuntary assimilation practice, Keetoowah youth were forced to attend boarding schools [58]. These AI/AN boarding schools did not allow students to speak their traditional languages or follow traditional cultural practices, and because students were separated from their parents and communities, they became victims of emotional, physical, and sexual abuse [58]. It has been found that the past and present culturally oppressive practices experienced by the Keetoowah people continue to have far-reaching impacts. The well-being of the UKB has been weakened by disintegrating cultural ties and adding cultural ambiguity, such as the feeling of not knowing their culture and being unclear about where they fit in culturally [24,58].

Before Western encroachment, the UKB and the Cherokee people were traditionally a matrilineal society, with the woman being central to the household [56,59,60]. Until settler colonialism upset the traditional gender roles of the Cherokee people, motherhood was the source of power and status, tied to the identity of women within Cherokee tribes. Historically, when a woman married, the man moved into her household, and children remained under the mother’s clan affiliation [56]. Blood was considered sacred and capable of altering wars, family life, and hunting for food, and as such, maternity was held in reverence, and menstruation and birth were regarded as a powerful time in a woman’s life cycle [56,59]. Because gestation was considered to be such a powerful time in a Cherokee woman’s life, she was restricted from activities wherein it was believed she had power over the ceremony or cultural event [60]. While a Cherokee woman was pregnant, she was showered with spiritual rituals meant to “guarantee safe delivery”, in which pregnant women drank elixirs containing elm bark. In addition, women avoided foods believed to place the baby at risk and consulted with tribal leaders for purification rituals [59]. The birthing process was very much a community event in which women from the tribe would attend the birth, as well as tribal healers and other medicine people who sang and aided in the birthing process [56,59]. Women birthed their children standing or squatting, wherein the baby was caught in a soft bed of leaves, rather than the hands of someone else [59]. After birth, women in the tribe aided in the mother’s recovery and aided in the development of the infant’s character [59]. Before colonialism, Cherokee women held autonomy within their sexual, pregnancy, and birthing practices [56]. 

During settler colonialism, White male settlers were astonished by the relative freedom and control Cherokee women exerted on their tribe and community [59,60]. True to colonial beliefs, White men worked to convince Cherokee men that because women held such power and autonomy, especially around control of the household and household management, marital arrangements, and childrearing, this was a challenge to their masculinity and power. By “allowing” women to take ownership of these areas of life, Cherokee men were waiving their “God-given” rights as men of the household [60]. 

Furthermore, the practice of polygamy within the Cherokee people was regularly accepted based on the Cherokee woman’s position in the tribe [59,60]. As such, the Cherokee practice of marriage was different from the strict, delineated puritanical standards of early settlers or the Westernized version known today in the US [60]. Even the concept of sin in the Cherokee tribes was less about individual morality and more about the whole of the tribe or activities that “upset the equilibrium” [60] of purity. During the Dawes Commission (1899–1907), all people of the “five civilized tribes” were set to be documented by the US government; Cherokee marriage practices came under assault and were found to be morally objectionable to puritan settlers. As such, congress was encouraged to outlaw their practices of polygamy and other “morally objectionable” marriage practices and force Cherokee tribes to define their marriages under the law [60]. This attempt was a further assault on Cherokee women’s traditionally held power as matriarch and sought to supplant Cherokee women as the head of the household to acculturate Cherokee people into a world where capitalism and patriarchy dominated [34].

### 1.5. Gaps in Research and Current Study

This historical process that Keetoowah mothers have undergone, wherein they came from a culture that empowered mothers and encouraged an egalitarian tribe that was then shifted into one that stripped them of their cultural ties to maternal role transition and power has influenced BAM generationally. However, no studies address BAM in Keetoowah culture, which is distinctive, homogenous, and influenced by historical trauma and reproductive injustice. Indeed, few studies frame maternal mental health challenges through reproductive justice and historical trauma frameworks to evaluate the culturally specific historical contexts influencing BAM for AI/AN mothers. This framing intends to recognize unique experiences of the BAM process for AI/AN mothers. As such, this study addresses the gap in the literature by acting as foundational qualitative research on tribe-specific expressions of BAM. Using BAM, historical trauma, and reproductive justice frameworks, it poses three research questions: (1) What are the experiences of becoming a mother and postpartum mental health among Keetoowah mothers? (2) How does Keetoowah culture relate to the postpartum experience for Keetoowah mothers? (3) How does historical trauma intersect with Keetoowah mothers’ postpartum experience?

## 2. Materials and Methods

This study used a story inquiry method to collect and analyze data from eight Keetoowah mothers between August and October 2020. Researchers obtained approval from the Institutional Review Board of a large university in the South–Central US and Keetoowah tribal support before any research activities. This project was funded by the (blinded for review), which had no role in collection of data, analysis, or approval of this finished manuscript.

### 2.1. Story Inquiry Method 

Since AI/AN cultures rely heavily on oral tradition and storytelling, the story inquiry narrative approach is a culturally appropriate qualitative methodology [61]. Story inquiry differs from narrative methodology in that it allows for participants to understand their place within a communal narrative. Story inquiry expands beyond individual narratives and ties in historical stories and contemporary experiences, while allowing participants to reclaim their communal story collectively [62].

Furthermore, storytelling allows individual participants to recognize their power and contribution to their community’s larger narrative and their ability to affect change [63]. Similarly, stories offer healing potential and relate to holistic human health by allowing people to organize their thoughts around a phenomenon or experience [62]. Stories also provide intricate details that are often lost within quick conversations or quantitative assessments, allowing story building to provide a unique and comprehensive understanding of what a population is experiencing. By allowing participants to express their experiences through stories, a storytelling approach is a culturally appropriate and decolonizing method and allows for the interviews to gather rich data to capture the essence of the phenomenon [61,62]. The story inquiry approach has also been used in prior research with the Keetoowah tribe [61]. Story inquiry method begins with gathering stories about a health challenge. Following the story gathering, deciphering dimensions of the challenge begin, and then a story with low and high points is developed. Then, the movement toward resolution is identified. Finally, the stories are synthesized to “address the research question” (p. 242, Reference [62]).

### 2.2. Research Site and Context

The research took place in Tahlequah, Oklahoma, and its environs. The UKB has its tribal headquarters in Tahlequah, a town with 15,753 people, 30% of whom are AI/AN. The headquarters includes an elder center, a childcare center, and a cultural museum. 

### 2.3. Recruitment and Sampling

This study was limited to mothers who are members of the Keetoowah tribe and who had given birth within the last two years, aged 18 or older. The birth requirement was placed on the sample in order to limit the possibility of retrospective memory issues. Because of the strict inclusion criteria, the sample was mainly recruited through both snowball and purposive sampling. Purposive sampling techniques included paper flyers, recruitment through social media platforms, and in-person recruitment in a civic location where UKB frequented. The vast majority of interested participants (*N* = 6) came from a single recruitment effort at a cultural event. Additional snowball sampling recruited two more mothers who fit the criteria for participants. All 8 of the mothers who agreed to participate signed informed consents and gave the researcher their contact information, including a time to be interviewed. The final sample consisted of 8 participants who completed interviews as the study reached data saturation at this point. Study participants were offered a $20 gift card for their participation.

### 2.4. Data Collection

Participants were given a choice to complete the interviews in any space in which they felt most comfortable, including by telephone. As such, all eight participants made a choice to be interviewed by phone, due to time constraints and childcare issues. Interviews were conducted by the first author, who is extensively trained in qualitative methods and has an existing research relationship with the tribe. A digitally encrypted recorder was used to record all phone interviews. In addition, informal talks with midwives and other service providers, such as social workers, occurred before and during the study; researchers took field notes during these instances for researcher reflectivity.

### 2.5. Interview Guide

There were 14 total questions on the interview guide. The average length of the interviews was 56 min. The questions asked in the interview were open-ended and were meant to elicit a story. For instance, the opening question was, “Would you please share with me your birth story?” Follow-up questions contained prompts which were meant to gain a better understanding of the birthing and postpartum experience and included questions such as the following: “If you could change the way people in your tribe go through pregnancy, birth, or taking care of babies, what would you change?” 

### 2.6. Data Analysis

Our analysis followed the established story inquiry data analysis method [62]. The first step was to transcribe the storytelling interviews verbatim. Once the interviews were transcribed, the stories were then read twice by the authors with breaks between to allow for immersion into the data. During these initial read-throughs, the authors documented thoughts, potential themes, and shared plot ideas. Only after reading each transcript twice did the coding begin, using atlas.ti (v.8.1) (Atlas.ti, Berlin, Germany). 

The transcripts were thematically coded by three of the researchers (one author did not participate in formal coding) for plot points and shared plots [62]. Plot points are aspects of a similar story and can be organized into the seven essential story elements: characters, setting, plot, point of view, symbolism, conflict, and resolution [62]. Then the same three researchers used atlas.ti (v.8.1) to organize thoughts, themes, high points, and low points of the plots. The story plot points were also coded for theoretical thematic content, specific aspects of the story relevant to historical trauma, and BAM since these are theoretical support for the study. These themes and visualizations were then triangulated with the researchers by consensus and with participants (mothers), midwives, social service workers, and tribal council. 

This study utilized all five steps of Synthesized Member Checking (SMC) to ensure the participant’s and the community’s voices were at the forefront of the findings. SMC is a five-step member-checking process. It ensures that participants can see the interview and interpret data to validate their experiences being reported in the study [64]. Following SMC’s tenets, researchers prepared a synthesized summary in non-scientific language for the participants with emerging themes and quotes. In the second step, all participants were asked if they were willing and in an emotional space to review the materials. In the third step, researchers sent a summary to participants with relevant questions, such as “does this match your experience?” and “do you want to add anything?” [64]. Five of the eight participants responded with additional feedback, agreeing with the thematic content and also addressing any information which could identify participants to other tribe members to be removed. Tribal council members and an elder were also provided with the thematic content and quotes and aided in redacting sacred information or potentially identifying information, but also agreed upon thematic content. In the fourth step, researchers added the responses to the data and removed potentially identifying information; then, in the fifth step, researchers integrated those responses into the findings.

## 3. Results

### 3.1. Sample Characteristics

The final sample of mothers had a mean age of 31.5 years. The modal level of education for the mother was “some college”. On average, mothers lived 20 miles from the Indian Health Services hospital, where they were most likely to receive prenatal care, give birth, and receive postnatal care. All of the participants lived or worked in the same primary geographic region of the state.

### 3.2. Themes

Within these findings, there is a flow of time, and stories sometimes slip from past to present to future, which indicates the connection between the three. The thematic demarcations are superimposed, an artifact of language limitations, and represent as best they can the place in the time being discussed. These findings described how the Keetoowah are here now and how they experience BAM currently, while also accounting for the past stories. In this present story, two primary themes, maternal mental health challenges and inadequacies of perinatal care, and their related subthemes describe the intersection of reproductive justice and historical trauma within the context of becoming a mother (see Table 1). 

Theme 1: Maternal Mental Health Challenges

- PPD was a salient aspect of BAM for the participants. This theme encompassed their experiences with PPD, the intersection of historical trauma with becoming a mother and their mental health, and their need to “be strong” in the face of mental health challenges was prompted by historical contexts.

**Subtheme 1: Prevalence of PPD Experiences.** Seven out of the eight participants discussed experiencing PPD during their most recent pregnancy. Some of them discussed having mental health challenges for as long as they could remember; others discussed being somewhat blindsided by PPD after their child’s birth. They discussed various experiences with PPD symptoms. One mother described her mental state in the postpartum period, saying the following:

“Oh my gosh. So, I’ve always kind of struggled with anxiety and depression. I think whenever you do that, whenever you have those issues, before you have a baby after you have a baby and your hormones are out of whack, especially, at least my experience was if you’re breastfeeding, my issues didn’t go away until I stopped breastfeeding. So, in hindsight, I wish I would have recognized or knew that earlier. I think I still would have breastfed as long as I did, but those first few weeks were hard. Mentally you’re all over the place”.

Others described that despite having people in their lives to support them, PPD still prompted them to feel isolated and lonely. One mother shared the following: 

“I would just get in my own head and think about stuff and second-guess myself and felt lonely even though I knew I wasn’t lonely. I knew a lot of people were there for me, but that’s just what it felt like to me”.

Another mother described that she felt like since her main support, her mother, was no longer living, she did not have anywhere to turn, saying, “*Well, my mom’s gone. She passed early. And so, I kind of slipped through the cracks. I kept, with this one, I had postpartum. And I hid it*”. 

This hiding was present in many of the stories, and the participants hid for various reasons. Some felt they simply did not have “time” to be depressed due to their hectic lives, such as this mother who said the following: 

“I did a lot of hiding; I did a lot of withdrawing. And finally, I had to come to terms with this could be my family that’s going to lose me, or I’m going to lose my family. So that and work, work stress, having a high stressful work environment. I just had to go with it. I had to go get on medication because I could, I was pretty close to suicide. After I came to senses, I realized okay, that’s not me. And I went and got on medication”.

Others expressed hiding their feelings by keeping them to themselves. For instance, when asked if she talks to people about her PPD, one mother responded, “*Not really. I just kept them to myself. I’m not big on expressing my feelings”*. That being said, others hid because they felt guilty feeling depressed because they have more economic and educational resources than other mothers in the tribe. This guilt prompted them to compare their suffering to others, ultimately ruling that they did not think that they had the “right” to be depressed. On the other hand, this recognition of privilege acted as a metric for them to know they needed help with their PPD. One mother exemplified this experience with withdrawal, hiding, and seeking help with her PPD when she shared the following: 

“I did because I realized… I live on 120 acres. I have a vehicle. My kids are taken care of. Why am I not happy? There’s so many other reasons, and I’ve been down the rough road with the other kids’ dad where they didn’t work. It was me doing all of it. And here I have someone that was doing all of it, and I still couldn’t be happy. So, I knew something was wrong with me”.

Knowing that many of their peers were not as financially secure or did not have as much access to education as they did prompted guilt that kept them from seeking informal and formal support and services. Even deeper, the mothers felt guilty for being mothers who had resources, which ultimately impacted the way they felt about motherhood and mental health. The intersection of historical marginalization among the tribe (such as having higher rates of poverty and substance abuse) and finding success within the Westernized world created guilt. 

**Subtheme 2: Historical Trauma Exacerbates Mental Health Challenges.** The past treatment of AI/AN peoples and, more specifically, mothers was not lost on the participants concerning their transition into motherhood. Many participants expressed that although they recognized that they had PPD, they felt the experience was deepened by recalling historical trauma and how even being an AI/AN mother was an act of painstaking resistance. They discussed how their cultural mothering practices and being denied the ability to be Keetoowah fully in their mothering were just a reminder of what had also happened to their ancestors. Additionally, the mothers expressed how the dismissive and substandard perinatal care and support they received, including a judgment for mothering within their culture, was a continuance of the perpetuation of oppression and erasure of their unique experiences with motherhood. When explaining why she thought PPD was such an issue to mothers in her tribe, one mother described how she felt it related to historical trauma:

“I think it all goes back to; I think most of the issues that we face today in Native communities all go back to, I mean you have to evaluate how Indians have been treated for the past 200 years. While we may not have felt that specifically today, one, our ancestors did, so if you have done any research on epigenetics, it’s literally imprinted on our DNA”.

Another mother supported this, relating that the historical grievances perpetuated against AI/AN people were the root of maternal mental health issues and that ignoring the past when evaluating mothers’ current status in the tribe was deleterious. She said the following:

“I think it all goes back to the intentional attempt to annihilate the tribes and tribal members. I mean, you can’t tear apart communities and strip people of their culture and expect that to go away in one generation. I think all of the, most of the issues we’re facing today can all be tied back to that. Obviously, we’re not holding people, necessarily holding people today accountable for stuff that happened in the past. But it would be stupid not to understand how past dealings are affecting today”.

Another mother discussed the incongruence AI/AN mothering had with Western-colonizer parenting styles and how that has prompted a dichotomous approach to mothering within her community, sharing the following: 

“Also, the effects of that are our communities now where we have higher rates of poverty, higher rates of unemployment. Parenting, traditional parenting, did not buy well with Anglo parenting. So, there was kind of a disconnect between what was appropriate and what was not appropriate. So, I think there’s also kind of a—I don’t know; I think we’re still feeling it today”.

Beyond the inability to mother traditionally due to cultural erasure and genocide, the mothers shared that historical trauma perpetuated by colonizers has created fragmentation in trust with care providers, ultimately impacting their access to perinatal care and reduces their reproductive options. Even more so, they felt that this historical oppression made it hard for them to want to disclose mental health issues. For instance, one mother explained the following: 

“Well, and it goes back to the disclosure and seeking help historically when you ’re told not to use your language or your culture of allowing your male child to wear long hair is inappropriate. So, we’re just going to cut it, and we’re just going to tell you, you can’t wear it. You can’t talk about it, you can’t speak your language, and you can’t go do your ceremonial dances because why should you keep children up all night long? Whenever they take that all away, why would then Indians go and try to seek help when every time they have, they’ve just been told, stop it. You’re doing it wrong anyways”.

Finally, the mothers expressed that they felt that historical trauma deepened the PPD symptoms they experienced, adding fuel to their anxiety, anger, and depression postpartum. In addition to having the more documented PPD symptoms, such as worrying about the baby, feeling isolated and lonely, and feeling like an inadequate mother, the mothers in this study felt angered by what had happened and still happens to their people. Sharing this, one mother said the following regarding her postpartum mental health, 

“I’m so angry all of the time. I’m not violent. I’m just generally angry. I mean, that was my thing. It was like I was so resentful, and I was just frustrated, and a lot of it went back to anxiety and depression and all that stuff. But even, I think even if our more affluent tribal members are afraid to admit that and are afraid to seek help because they’re afraid of the consequences, what do you expect someone with maybe a high school education who probably has a criminal history who can’t keep a job, what do you expect them to do?”

Despite experiencing PPD symptoms that seem universal, the participants had added symptoms, including anger and anxiety arising from historical marginalization. This added grief impacted their motherhood, as it illuminated for them the juxtaposition of their motherhood experience with others in their tribe and sometimes created guilt. 

**Subtheme 3: We Have to Be Strong.** Beyond historical trauma, the historical context of Keetoowah matrilineal society impacted the participants’ experiences of becoming a mother as well. Even with assimilation, their ancestors’ values that insisted that women run the household remained salient for the mothers. The need to be the rock of the family promoted a silence of PPD symptoms and distrust in providers to handle them appropriately. One mother described how the cultural pressure to be a superwoman kept her from seeking support:

“Maybe the hormones or whatever is totally different. It took more of a toll on my body. It was harder; it just hurt more. I was more tired. And so, I realized I wasn’t a superwoman like I was with the first, second, or third. I had to learn to; it’s okay to take maternity leave, it’s okay to be at home. But I had, having a great man helped to alleviate some of that stress”.

Similarly, even when friends and family outright recognized the symptoms of PPD, mothers discussed how they remained steadfast in their appearance of being the strong mother. When describing her dismissal of family noticing her PPD and how people in her culture do not discuss mental health, one mother shared the following:

“My dad would come over, and I would be sleeping or something. He’d be like, ‘Are you all right?’ I’m like, ‘Yeah. I’m fine’. He was like, ‘You don’t have postpartum, do you?’ I was like, ‘I don’t think so. I think I’m just tired’”.

Meanwhile, other participants recognize that the stigma of mental health challenges among AI/AN people is shifting, even if slightly. This shift allowed them to give themselves some grace, even if they still did not admit their symptoms. Another aspect that contributed to their silence was their standing in the close community of the tribe. The participants felt that if they were more involved in the tribal government or were in high community standing, it was harder to disclose their mental health challenges because they needed to present as strong mothers heading the household that much more. For instance, one shared the following:

“And we’re kind of in a more of an era now that it’s okay to say, but still at the same time … whenever you have a high stressful job, or you’re in a spotlight … so we have to be careful. So, I think our reputation sometimes, depending on your reputation, you have to kind of keep some things under wraps. I think those were some of them. But I think maybe I was allowed to get a little bit; I think with more support, I wouldn’t have got in deep”.

Furthermore, they again discussed how AI/AN people’s historical treatment had left them unwilling to share their feelings or trust non-AI/AN providers, resulting in a cultural stigma of talking about mental health in general. One mother summarized the hesitance to discuss PPD within her cultural circles, saying, “*I don’t think it’s [PPD] talked about a lot. I think that there’s kind of the sentiment where, well, I can just take care of it on my own. I don’t need help or anything like that”*. Another reinforced the resistance to sharing about mental health, revealing, “*Okay, so I get medication, but I think the Indigenous part of me doesn’t want to talk to anybody because I know that they’re not going to understand”*. Others related that the lack of disclosure of mental health challenges was a feature of being AI/AN, with statements such as, “*A lot of Native people are more keep everything balled in*” and “*Native people are more stubborn, like to come off as tough and like nothing can get to them*”. This self-described stubbornness leads to internalization and a lack of seeking support for PPD symptoms. When asked what perinatal care providers in their community can do better, one mother reported that she felt that AI/AN mothers experienced PPD more frequently, saying the following:

“There certainly should be a focus on it [PPD] because I know it’s something I struggled with. I don’t know. It’s funny because I know that my non-Indian friends, some of them struggle with it too, but thinking about it, it’s like those moms who are just fine and carry on with their lives, and they’re able to go on trips with their baby. It’s almost as if having a baby didn’t affect them. But then I think about all of my Indian friends, and I really reflected on it. It’s like man; I don’t know any who have responded that well or at least pretended to respond that well to having a baby. My Indian friends really struggle with it”. 

Additionally, the resistance to disclosing PPD arose out of a desire to remain true to traditional practices that do not involve “Western” medicine and use traditional medicine to support mothers experiencing mental health challenges. Some mothers felt that by telling providers about their PPD symptoms, they would be given psychopharmaceuticals and not be encouraged to engage in traditional medicine they felt was necessary to heal. One mother summarized this, saying, “*I hid it [PPD] for a long time because I felt less than. I’m still trying to get that under wraps as we speak. And being ceremonial, you don’t want to act, I rarely turn to medication*”.

Theme 2: Inadequacies of Perinatal Care 

Following the theme discussing unique maternal mental health experiences for Keetoowah mothers is the present story of perinatal care. Within this theme, there were subthemes of wanting providers to focus on them and the baby once they were born. Another aspect of perinatal care heavily present was the second subtheme: the constant changing of care providers, which precluded developing a trusting relationship. The third subtheme centers around not being able to listen to their bodies during the perinatal process and providers’ inability to recognize the legitimacy of cultural values surrounding birth.

**Subtheme 1: Supportive Focus on Mothers, Please.** The first subtheme discussing perinatal care in BAM stories for the Keetoowah women is that they wished that perinatal providers would focus on them in addition to the newborn in the wake of birth. Many women felt that they were figuratively and literally pushed aside and forgotten about by providers once they had given birth. Furthermore, they felt as though the care they did receive was dismissive and lacked transparency. This dismissiveness prompted the mothers to feel as though providers took on a paternalistic role that diminished their capabilities as mothers, prompted them to have reduced maternal confidence, and resulted in a lack of trust in their providers. For instance, one participant related the following: “*And one of the midwives was just dismissive, and she’s like,* ‘*Oh, you’ll be fine’. Like, no, that’s not answering my question. I want you to give me an answer*”.

Another mother described how the child needs to be seen multiple times after the birth; however, the mother has limited appointments to address concerns, particularly mental health concerns that may arise as they temporally move further away from the birth. She shared the following:

“So, I went two weeks and then six weeks and then that’s it. I haven’t been back to the doctor. So, I think there’s kind of a discrepancy in that there’s so much focus on the child once the baby is born, which is needed. I’m not saying it’s not needed. They go back for their one-week appointment, their two-week appointment, their two-month appointment, their four-month appointment, their six-month appointment. And I think once women are past that six-week mark or whatever, then it’s like, all right, have a great time. And there’s not really follow-up with that”.

Some of the participants who felt they had more education than their peers felt that providers acting dismissive toward mothers could impact those with less education even more than they themselves had experienced. They felt as though providers not listening to them and assuming that they did not know anything about the childbirth process could lead to negative consequences, particularly for those who did not feel as though they had a voice when interacting with providers. Similarly, they expressed that less educated mothers may not know their prenatal testing options and birthing plans. The lack of provider transparency about such options could lead to these mothers having issues. One mother shared the following: 

“It really, really concerns me [lack of provider transparency relating to less educated mothers]. In fact, after I had my baby, I sat down with the doctor who is over the department because he was my doctor, and I was like, ‘I have a lot of concerns’. And I meant to write a letter, and I never got to it. But one of the concerns I had was the blood test that you get to determine whether or not your child has downs [Down Syndrome]. I wouldn’t have had that done, but for my persistence. I said, ‘When am I supposed to get this taken care of?’”

**Subtheme 2: Revolving Door of Providers**. All of the participants described their frustrations with Indian Health Service (IHS) regarding not maintaining the same perinatal care provider throughout their pregnancy and birth. The mothers described how providers’ revolving door prompted uncertainty for them and continually led to inconsistent information from perinatal providers. For instance, one mother said the following: 

“So again, every time you go, you get a different midwife. But some of them were very dismissive of my questions… So, I would always ask the same question like, ‘Okay, when is my next ultrasound? Okay, what about circumcision, because I found out I’m having a boy’. And there were no consistent answers across the board”. 

Another mother insisted that the inability to have a consistent perinatal care provider is what prompted her not to like IHS care, saying the following: 

“Yes. I mean, for the most part, yes. So, the way I don’t like the IHS visit is, every time you would go to your prenatal appointments, you would have a different midwife that was there”.

Others described how not only was the lack of a consistent provider frustrating because of the differences in information and care received, but they also felt as though it inhibited them from being able to form a relationship with the provider, meaning they did not get an opportunity to build trust with the person who was providing perinatal care. One mother related the following: 

“So sometimes you just get whoever’s there. And I think that you lose the sense of a relationship. You have to have sort of trust and relationship. They’re fixing to get real up close and personal with you”.

This trust was essential because it provided comfort for the mothers; it allowed them to express their culturally relevant traditions regarding BAM. Without this, they either were left feeling exhausted from repeating themselves or abandoned their traditions altogether because the inconsistency in care disrupted their ability to communicate their traditional desires. One mother explained this, saying the following: 

“I think because I personally, this was the third child that she [healthcare practitioner/midwife?] delivered, so she was well aware of my ceremonial requests, and she was more aware of me personally. I think sometimes when you go to [IHS hospital], or you get, you go to those places, you’re never stuck with one person”.

The other issue with the rotating door of providers that influenced trust and ability to communicate about their cultural birthing preferences was the chance that they might get providers who outright dismissed their cultural traditions. The mothers described how they wished they could have the same provider to build trust, explain their cultural practices, and practice these without judgment. Many of the mothers discussed that providers knew how inconsistent they were, as one said the following:

“They just didn’t seem to care. They were dismissive. There was one midwife who kind of talked crap about the other midwives and were like, ‘Well, you better hope that you have her because I won’t do that’. And she was like, ‘If I have you, then this is when you can have your epidural’. So, it was personality conflicts in a way. And I don’t think they were used to having someone who asked as many questions as I do”.

Unfortunately, many of the mothers expressed being shamed over their cultural practices. Their birthing and new mothering approaches were not taken seriously and were often visibly frowned upon by providers. One participant mentioned, 

“Of course, that’s hard [living traditionally] in the modernized world, but as far as giving birth to my kids, I, let’s see, I had to have Cesareans. And so, with my kids, I kept the placenta and buried it afterwards. And so sometimes through that, you have doctor’s kind of look at you weird and kind of want to ask questions, but understand at the same time that I’m not obligated to answer. And so out of niceness, I give them a little bit of information; I just don’t ever go too much into detail”.

Overall, providers’ revolving door meant that the mothers did not feel trust for the providers, lacked rapport with them, and did not feel comfortable or felt exhausted from sharing their cultural traditions. Since providers were always changing, the women did not get consistent answers and missed out on prenatal tests, which could mean they would have children with health issues.

**Subtheme 3: A Colonized Birth Experience.** The women felt as though other reproductive choices were foreclosed, primarily regarding how to bring their baby into the world. The participants described how they felt as though there was no room for dialogue on their reproductive choices and how this lack of ability to interface with providers made them feel frustrated with the entire endeavor of giving birth. Even though they recognized there was merit in needing medical intervention such as cesarean sections (i.e., C-section, surgery to deliver a baby through the abdominal wall) and induced labor, they felt like the rate was too high within their community and that it had to do with the inability of providers to listen to them. The mothers also expressed how they felt as though this inability to express their birthing desires was another historical trauma aspect. The colonizing doctors just wanted to do things their way and ignored the needs and cultural differences of the AI/AN community.

The participants described provider responses that often relayed a message that if they were American Indian, they would have to have a cesarean. One mother described said the following: 

“But I think that’s just kind of the standard practice because there’s the potential for risks during labor and delivery. Instead of evaluating that on a case-by-case basis, they’re kind of just making blanket rules where if you haven’t had the baby at this point, you’re getting induced. If you have this, this, this going on, you’re getting a C-section and just kind of really getting away from the more natural doing what our bodies were made to do type of thing”.

Others explained that doctors outside of the tribal hospital were more willing to listen to them and that the IHS hospital providers were aware of the bias and did not seem to care. One mother described a doctor’s response when she went to a well-baby check at the IHS provider after giving birth elsewhere: 

“When we had our first visit [at the tribal hospital, not where the child was delivered], their doctor said straight up, ‘If you would’ve had her here, you would’ve had to have a C-section. You wouldn’t have been able to have her vaginally’. So, the rates of induction, the rates of C-section, the rates of antibiotics during pregnancy, I think all of that is higher than what it would’ve been historically. There’s obviously a need for that in some circumstances”.

Even more impactful was that, when they did share their cultural traditions, they felt judged and dismissed, so they often went without practicing them altogether. This systemic dismissal of AI/AN mothering practices further enforces the erasure of their motherhood and the reproductive options they might have. 

## 4. Discussion 

These mothers’ shared stories highlight many poignant aspects of BAM among Keetoowah mothers. When framed through both the historical trauma and reproductive justice frameworks, the findings in this study highlight the necessity of addressing how some Keetoowah mothers have historically been denied access to their motherhood, which is not only inhumane but also serves as another agent of cultural erasure and genocide and contributes to maternal mental health challenges. This study addresses a gap in knowledge regarding AI/AN motherhood and maternal mental health and can inform future research on PPD and maternal mortality.

Due to many historical contexts, such as the increased prevalence of substance use and lack of educational opportunities, participants expressed a lack of transparency around the method of delivery. Providers often assume that AI/AN mothers will need a C-section or induction, often failing to listen to the mother and her wishes to birth differently if possible and if it has no potential impact on the infant’s health. The participants in this study describe a persistent inability of providers to listen to their personal experiences instead of assuming medical interventions based on racial/ethnic identities. Moreover, a legacy of White-centered cultural values surrounding pregnancy and birth that has delegitimized AI/AN mothering practices influenced the birthing experience for the participants in this study and ultimately impacted their mental health. Invalidating AI/AN birthing practices, such as placenta burying, highlights the ways perinatal care providers perpetuate White supremacy within birthing realms. This delegitimization of AI/AN mothering is inhumane and serves as another agent of cultural erasure and genocide. Furthermore, the erasure of AI/AN motherhood is compounded by other historical inequities to exacerbate PPD symptoms, most notably in this study, as anger toward historic treatment arose in the postpartum period in conjunction with other PPD symptoms. These findings elucidate the necessity for traditional approaches to be supported by existing healthcare services and the need for culturally developed healthcare approaches.

Attempts to colonize mothering and birthing go beyond delegitimization of birthing practices and extend to cultural erasure. Participants in this study described how the inability to access cultural knowledge surrounding BAM, such as medicine men who name children or knowing what their ancestors did during this transitory time, influenced and exacerbated sad feelings experienced by them in the postpartum period. Mourning the loss of the pre-child identity is one aspect of BAM, and the addition of mourning cultural loss (highlighted during this personal transition) aggravates PPD symptomology. This aspect of BAM and maternal mental health is unique to a group experience with the intersection of historical loss and persistent reproductive injustices resulting from continued colonization. 

Overall, some PPD symptoms for the participants were similar to existing research in that they felt isolated and lonely. Additionally, culturally specific needs to “be strong” for the sake of the community and their families promoted enhanced loneliness and lack of desire to disclose their PPD symptoms. This sentiment is mirrored in the existing literature as well. For instance, in Knudsen-Martin and Silverstein (2009) [65], internalized fear of isolation, as well as the pressure of idealized motherhood, prompts those with PPD to withhold their experiences.

The women in this sample were primarily caregivers of their families and community, often expressing concern for those with less education and resources. The findings also highlighted survivance and resiliency born within AI/AN communities out of the necessity of communal action and connectivity. 

## 5. Limitations

The results of the current study should be interpreted in the context of the limitations. The participant qualification of being enrolled in the UKB, giving birth within the past two years, and being over the age of 18 substantially narrowed the potential pool of participants. Although many new mothers were identified within the tribe, many were under the age of 18, precluding their participation. Furthermore, considering the ruralness of the location and the tribe, it is to be expected that potential participants may not have been at the main cultural event where recruitment took place. As such, the diversity of the sample size is less broad than originally targeted. 

Because of the aforementioned limitations, the study lacks demographic diversity. There was an expectation that ethnic diversity would be limited, as the sample was restricted to Keetoowah members. However, there was also an expectation that there would be diversity in education levels. That said, because of the ruralness of the tribe and its location, more relatively “affluent” women were enrolled in the study, with most having “some college” education. Because of this, it is likely that the study did not include mothers using the child welfare system or other social services or receiving substance-use treatment. We expected that these experiences would lead to a unique experience of BAM and may not be represented in the current findings. Although the demographic region was rural, the participants themselves did not live in a rural community, meaning that it is likely that their BAM experiences are only generalizable to those living in more suburban/urban areas or those living in areas closer to the IHS hospital. 

## 6. Implications for Practice

The study demonstrates the need for health professions to engage actively and maintain a commitment to increasing the number of maternal mental health specialists and providers. Specifically, this study shows the importance of the need to have perinatal and postpartum mental health providers who are culturally responsive. Because health professionals often work in a multidisciplinary team in the healthcare setting, it is crucial that their role includes advocating for culturally derived care and culturally appropriate practices for AI/AN women. Moreover, it is recommended that mental health and healthcare educators increase focus on maternal mental health within human behavior coursework and address maternal mental health issues in practice classes more fully. Furthermore, it would behoove practitioners to focus their state and national advocacy efforts on addressing reproductive justice and expanded reproductive rights and services that can enhance the access that AI/AN mothers have to reproductive services. 

To achieve such advocacy efforts, we recommend that schools educating professionals place a stronger focus on recruitment of AI/AN students, notably ones that can provide training and internships within the students’ home communities. By creating these opportunities for education and experience, there may be increased access to maternal mental health providers who are culturally aware of the unique challenges AI/AN mothers face during their experiences of BAM and PPD. More generally, a focus on culturally respondent care for perinatal providers such as social workers, doulas, midwives, OBGYN’s, labor and delivery nurses, and pediatricians can enhance the BAM experience for AI/AN mothers and may reduce deleterious PPD effects through potential primary prevention. 

Mental health professionals must address maternal mental health disparities among AI/AN mothers through continuous acknowledgment of the historical and ongoing role of mental health professions in colonizing that such professions have adhered to. By acknowledging how mental health professions have been involved in the denial of reproductive justice to AI/AN communities, through engaging in non-culturally relevant care, by involving the child welfare system at disparate rates, or by not addressing reproductive rights directly as a primary example of health disparities for ethnic minorities, such professions can begin to craft a research and education agenda that is responsive to the unique needs of AI/AN people. Because of the guardian/ward relationship that the federal government has over tribes, the federal government has provided free healthcare to AI/AN tribal members, called Indian Health Services. As a federally recognized tribe member, the member may go to any IHS facility in the United States to receive free healthcare. IHS facilities can be federally run, or, under Public Law 638, the tribe itself can manage the facility. In Oklahoma, where the Keetoowah is situated, there are approximately five IHS hospitals and numerous clinics. Of the five IHS hospitals in Oklahoma, two are maintained under federal management, not tribal, through the 638 programs. However, the birthing process may only be at a hospital (One of the contributing authors is a United Keetoowah Band member and attorney for the tribe and contributed to these definitions). 

Since AI/AN mothers cannot birth their children in the ways they want, are not able to receive perinatal testing they want, and are not able to mother the way they want (burying placenta, washing the baby in the river, forgoing C-sections and inductions, etc.), their reproductive rights are continuously denied. Additionally, through acknowledgment, mental health professions can begin to engage in more dialogue for transformative healing surrounding historical trauma. Such dialogue provides numerous opportunities to ensure that advocacy efforts are in place not to overlook AI/AN mothering since such dismissal leads to further cultural erasure and infringement on their reproductive rights. 

## 7. Implications for Future Research 

This study’s findings illuminate numerous implications for research. Since this study focused only on a small sample of culturally homogenous mothers, many other populations within the United States may have similar barriers, such as lack of transportation, poverty, and historical-trauma impacts, which should be investigated to create more generalizable findings and to expand this study’s reach. In the same fashion, future research focusing on the BAM experiences of AI/AN teen mothers would increase our understanding of the unique etiological forces contributing to such high rates of PPD within this population. To that end, evaluating provider perspectives toward maternal care with AI/AN populations may illustrate potential interventions to address this mental health disparity. Evaluations of provider racism toward AI/AN mothers may also illuminate essential aspects of perinatal care that contribute to maternal mental health challenges. Research that appraises any statistical relationships between perceived racial stress and low birth weight or early term birth for AI/AN mothers can also provide insight into unique forces impacting AI/AN maternal mental health. 

Provider racism illuminates how historical trauma contributes to PPD. Furthermore, research exploring how specific tribal groups of AI/AN mothers score on the Historical Loss Scale [66] and the Historical Loss Associated Symptoms Scale [66] in relation to their scores on the Edinburgh Postpartum Depression Scale [67] might be of interest to quantitatively assess the relationship between the two. Beyond that, more research is also needed to evaluate the effectiveness of a community-based culturally relevant PPD intervention that utilizes the coping and spirituality described in this study. Future research on interventions that increase socialization and kinship among those with similar cultural and spiritual values can add to the knowledge base on culturally relevant PPD interventions and may decrease PPD symptomology for these populations. 

## 8. Implications for Health Promotion

In addition to the practice and research implications, the study’s findings also include health promotion implications as well. For instance, at the primary level, interventions which promote cultural birthing practices at the beginning of pregnancy can potentially promote buffers to developing maternal mental health disorders. Perinatal providers have a unique opportunity to create culturally appropriate care which can support AI/AN mothers from the outset of pregnancy. 

Furthermore, due to the high rates of PPD for AI/AN mothers, secondary prevention can be promoted through more consistent, culturally appropriate, and thorough maternal mental health screenings. Screenings which use tools that have been validated for AI/AN populations that account for culturally specific expressions of maternal mental health disorders, as well as increased frequency of screening among and within agencies who serve AI/AN women, will likely have impacts on earlier treatment and support. 

Finally, providers of mental health care to AI/AN mothers experiencing maternal mental health challenges can provide culturally appropriate care through becoming aware of historical harms and the impacts of those harms on specific maternal mental health experiences. Such tertiary approaches have the potential to mitigate some of the generational harm, while promoting culturally relevant and supportive mental health practices.

## 9. Conclusions

This current story of the Keetoowah mothers maintains some of the cultural heritage infused into the experience of BAM for Keetoowah mothers; however, their motherhood has been shaped by forced relocation, land allotment, marginalization, poverty, limited educational opportunities, high rates of substance use, and infiltration of Euro-centric technocratic perinatal care which denies them the ability to maintain their cultural mothering values. That said, the cultural values these mothers have been able to retain have been a source of resilience despite colonization, and cultural spirituality and traditions offer strength and support for Keetoowah women. This resilience allows them to look toward the future and maintain their values of ensuring their people have access to their culture and community and the strengths that come with that. Perhaps a hopeful takeaway is that this strengths-based approach indicates that the AI/AN motherhood role encompasses the necessity of transmitting cultural values, strengths, and resilience.

## Figures and Tables

**Table 1:** Themes and subthemes were identified in story inquiry research of the perinatal experiences of Keetoowah mothers (*N* = 8).

Maternal Mental Health	Inadequacies of Perinatal Care
Prevalence of PPD experiences Historical trauma exacerbates mental health challenges We have to be strong	Supportive focus on mothers, please Revolving door of providers A Colonized birth experience

## Data Availability

The data presented in this study are available upon request from the corresponding author. The data are not publicly available, due to the inclusion of protected cultural wisdom that may have been stricken from the findings and per tribal request.
